# Intussusception—Distensile Enemata

**Published:** 1875-01

**Authors:** B. C. Smith

**Affiliations:** Cold Water, Ga.


### INTUSSUSCEPTION—DISTENSILE ENEMATA.

By B. C. SMITH, M.D., Cold Water, Ga.

Some months ago I noticed a report, in the Journal, of a discussion on the merits of the forcible injection of water into the bowels, in cases of intussusception, and thought at the time that I would report my recent experiments in that line, but the matter was neglected; so I will now only give you a brief statement, because I think the results, in some degree, strengthen your position.

The first case occurred in a young man, while under treatment (with small doses of calomel and dovers powder) for acute dysentery. After using water enemas in a moderate way, until I thought the time had arrived for dividing responsibility, I communicated the patient's condition to Dr. Clarke, stating that I had not used the syringe *heroically.* He promptly advised its use to "the extent of overcoming the difficulty." I accordingly pumped water into the bowels until the patient could not be persuaded to bear any more; then let him rest a little, without permitting any to escape, and continued pumping until he exclaimed, "Doctor, you are bursting my guts!" I then gave another short period of rest, occasionally throwing in a little more water, as the bowels seemed to become capable of containing it. This state of distention was kept up about a quarter of an hour, when the water was allowed to escape. In about two hours thereafter the patient was able to defecate, upon which he expressed his gratification by saying, "That's worth half a dollar!"

The second case occurred in a man of middle age, while in good health. On the day previous to the accident, he had eaten peaches, for the first time during the season. The fruit was of excellent quality, and he experienced no difficulty from it until the following day, when he had an untimely movement of the bowels, followed by symptoms of intussusception. In two days time the difficulty had become serious, and I treated him as I did the first case—repeating the injections several times from midnight until daybreak. The last one I gave was pushed until he was in agony. After a few moments of rest, I asked him if he could bear any more. He replied, "if it was the only means of saving his life, he would try." The vomiting previously had only been of a bilious character, but at this time he vomited matters having a decidedly fecal odor. Being alarmed at this feature, I called counsel, but nothing more was required than about three hours rest, when his bowels acted naturally.

I have subsequently tried the same means in one case of *strangulated inguinal hernia.* No relief being obtained, the case was treated in the usual manner recommended in the books, except that no operation was performed, that resort being considered unnecessary on consultation, and it was postponed until it was found that a fatal termination was imminent, when the treatment was narrowed down to the hypodermic use of morphine. The post mortem revealed extensive adhesions between the hernial sac, omentum and bowel. One of the physicians who examined the condition remarked: "Mott himself could not have operated successfully."

The patient had never suspected the real character of the tumor, and even denied its existence on my first visit. On my second visit, eight hours afterward, I repeated the inquiry with much earnestness, when he admitted that he had a "swelled kernel, that always inflamed when he took cold, but he knew that it had nothing to do with his spell of colic." Like the majority of patients, he supposed that the portion of his ailments which he himself understood was not material to the physician.

As an illustration of the manner in which distension of the bowel relieves intussusception, I will mention that a circle of irritated hemorrhoidal tumors, in a state of protrusion and congestion, will be readily drawn in by distending the colon with water. This is not proposed as a plan for removing the tumors, however, as they return with the water.

